# Trends, advances, and emerging insights in hematopoietic stem-cell transplantation and viral-infection (1980–2024): A bibliometric analysis

**DOI:** 10.1097/MD.0000000000046814

**Published:** 2026-01-09

**Authors:** Minjing Mao, Jiacheng Zhu, Gang Cai, Jun Meng

**Affiliations:** a Department of Laboratory Medicine, Ruijin Hospital, Shanghai Jiaotong University Medical School, Shanghai, PR China.

**Keywords:** bibliometrics, hematopoietic stem-cell transplantation, immunotherapy, prognosis, research trends, risk assessment, virus diseases

## Abstract

**Background:**

Hematopoietic stem-cell transplantation (HSCT) is a cornerstone treatment for hematologic and autoimmune diseases, yet viral-infections remain a major complication affecting patient outcomes. This study aims to systematically map the research landscape of HSCT and viral-infections through bibliometric cluster and burst analyses, with the goal of identifying critical research hotspots and forecasting future directions for the field.

**Methods:**

A literature search was conducted in the Web of Science Core Collection database, covering publications in the research fields of HSCT and viral-infections. Bibliometric analysis and visualization were performed using VOSviewer, CiteSpace, and the R package “bibliometric.”

**Results:**

This study included 3247 publications, with the United States leading in output (1230 articles). Université Paris Cité was a prominent institution, contributing 112 publications. Blood was the most influential journal in the field, and Catherine M. Bollard was identified as a core author. Keyword cluster analysis revealed 4 main thematic clusters: clinical outcomes and risk management, basic science and mechanisms, immune response and immunotherapy, and viral detection and monitoring. Citation burst analysis indicated that recent research hotspots include “reactivation,” “management,” “mortality,” “risk,” “prevention,” and “impact.”

**Conclusion:**

This bibliometric study elucidates the dynamic evolution of research on HSCT and viral-infections, which has shifted from fundamental immunological mechanisms to patient-centered strategies emphasizing risk assessment, early diagnosis, and personalized management. Keyword clustering and emerging citation bursts provide evidence-based insights into future research priorities, underscoring the critical role of advanced monitoring, precision immunotherapy, and targeted prevention in optimizing long-term HSCT outcomes.

## 1. Introduction

Hematopoietic stem-cell transplantation (HSCT) has emerged as a pivotal therapeutic modality for the management of hematologic malignancies, selected hereditary blood disorders, and immunodeficiency diseases, demonstrating substantial efficacy in clinical outcomes.^[1]^ By transferring healthy hematopoietic stem-cells, HSCT facilitates the reconstitution of both the hematopoietic and immune systems, offering a viable therapeutic option for hematologic malignancies such as leukemia and lymphoma, as well as for nonmalignant hereditary blood disorders, including sickle cell disease.^[2,3]^ This modality has provided patients with the potential for long-term remission and has substantially enhanced survival rates.^[4]^ Nevertheless, despite its extensive clinical application over several decades, HSCT continues to face significant challenges, including delayed immune reconstitution and heightened susceptibility to infections, particularly viral-infections.^[5]^

Viral-infections represent one of the most significant complications associated with HSCT. The immunocompromised status of transplant recipients predisposes them to these infections, which are primary contributors to both morbidity and mortality in this patient population.^[6]^ Recent studies indicate that the incidence of clinically significant viral-infections after allogeneic HSCT can range from 30% to 70%, depending on the type of transplant, conditioning regimen, and the degree of immunosuppression.^[7–9]^ Among these, cytomegalovirus (CMV) reactivation is one of the most prevalent, affecting up to 60% of allogeneic HSCT recipients and approximately 10% to 30% of autologous HSCT recipients.^[7,10]^ CMV disease alone has been reported to contribute to a mortality rate of 20% to 35% in severely immunocompromised patients if not promptly treated.^[11]^ Other viral pathogens such as adenovirus, Epstein–Barr virus (EBV), and respiratory viruses (e.g., respiratory syncytial virus, influenza, parainfluenza) also contribute significantly to infectious complications post-HSCT. For example, respiratory virus infections can occur in up to 40% of HSCT patients, with associated mortality rates ranging from 10% to 30% in high-risk groups.^[12,13]^ The high prevalence and severity of these viral-infections underscore the complexity of their management and highlight the pressing need for effective preventive and therapeutic interventions.^[14]^ Therefore, it is crucial to gain an in-depth understanding of the current status and evolving trends of viral-infections in HSCT research.

Bibliometric analysis quantitatively evaluates scientific literature to reveal research trends, collaboration networks, and emerging hotspots within a given field.^[15]^ Although previous bibliometric studies have separately focused on HSCT^[16]^ and viral-infections,^[17]^ a comprehensive analysis of the intersection of these 2 areas is lacking. Therefore, this study aims to systematically analyze the knowledge structure, developmental trajectory, and collaborative networks of research on viral-infections associated with HSCT using advanced bibliometric methods. By identifying influential authors, institutions, journals, and thematic trends, this analysis provides a comprehensive overview of the current research landscape and evidence-based guidance for future research directions.

## 2. Materials and methods

### 2.1. Literature search and selection

The literature search was conducted using the Web of Science Core Collection (WoSCC) database, a widely used, comprehensive, multidisciplinary database known for its high-quality scientific research indexing.^[18]^ Based on a review of relevant literature,^[16,19,20]^ the final search strategy was formulated as follows: (TS = (((“bohematopoietic stem cell*” OR “hematopoietic stem cell*” OR “bone marrow*” OR “bone marrow cell*”) AND (transplant* OR grafting*)) OR “HSCT*” OR “allogeneic hematopoietic stem cell transplant*” OR “autologous hematopoietic stem cell transplant*”)) AND TS = ((Viral and (Infection* OR Disease* OR Illness* OR Contagion* OR Affliction*) OR “Viral Pathogen Infection*” OR “Virus-caused Illness*” OR “Viral Outbreak*” OR “Viral Epidemic*” OR “Viral Pandemic*”)). Only articles published in English were included. To ensure data consistency, all search operations were completed on September 2, 2024. This study analyzed only publicly available bibliometric data and did not involve human participants, patient records, or animal subjects. Therefore, ethical approval and informed consent were not required.

### 2.2. Statistical analysis and visualization

Bibliometric analysis and data visualization were conducted using VOSviewer (version 1.6.20), CiteSpace (version 6.3.R1), and the R package “bibliometrix” (version 4.3.3). VOSviewer was used to construct maps of collaboration networks.^[21]^ where node size indicates publication volume and line thickness represents the strength of collaboration or co-citation. Different colors were used to distinguish clusters and time periods, illustrating the evolution of research topics within the field. CiteSpace was employed to perform keyword burst analysis from January 1994 to September 2024 and to visualize research trend dynamics.^[22]^ The time slice was set to 1 year, and keywords were used as node types. PathFinder and network merging techniques were applied to generate a keyword timeline map for the field of “HSCT and viral infections.” The R package “bibliometrix” was used to calculate bibliometric indicators for leading authors and institutions, including the H-index, G-index, and M-index. The H-index measures both productivity and citation impact.^[23]^ The G-index assigns higher weight to highly-cited publications, highlighting those that have made the greatest contribution to the field.^[24]^ The M-index reflects the temporal consistency of academic output.^[24]^ Journal impact was evaluated using the IF and the Journal citation reports quartile ranking. The IF measures the average number of citations to articles published in a journal over the past 2 years, indicating short-term impact,^[25]^ while the Journal citation reports quartile ranking divides journals into 4 categories (Q1 to Q4) based on their impact, helping to assess their relative reputation and influence.^[26]^

## 3. Results

### 3.1. An overview of publications

**Figure 1.**
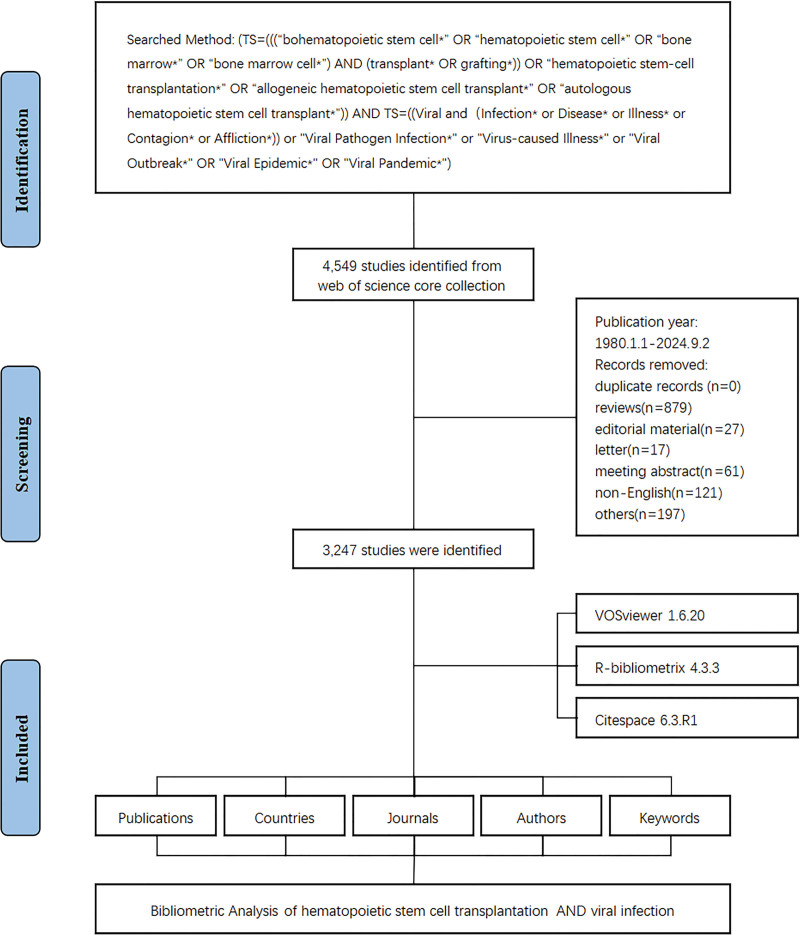
Literature screening flowchart. A stepwise diagram showing identification, screening, eligibility, and inclusion of studies for this bibliometric analysis. (Source: Web of Science Core Collection; Microsoft PowerPoint).

**Figure 2.**
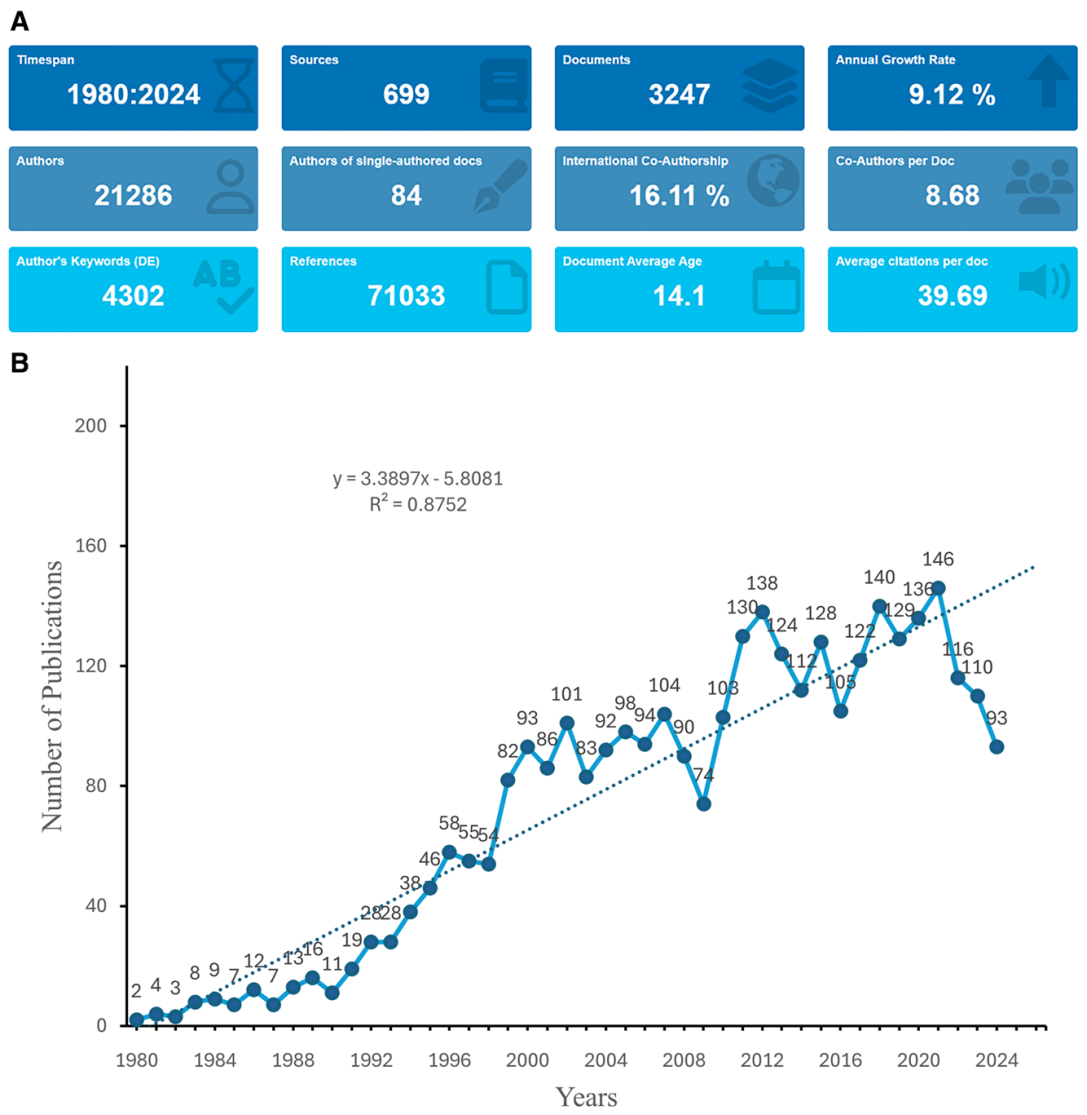
Overview of publication trends and study inclusion. (A) Overview of included studies by publication count, author number, and journal distribution. (B) Annual publication counts from 1980 to 2024, illustrating research growth over time. (Source: Web of Science Core Collection; R software).

The data selection process is illustrated in Figure 1. This study included 3247 publications on HSCT and viral-infections, covering the period between January 1, 1980, and September 2, 2024. A total of 11,858 institutions and 21,286 authors contributed to the relevant research. These studies were published in 699 journals (Fig. 2A). The peak year for publications was 2021, with 146 papers. From 1980 to 2024, publishing activity generally increased. Although growth was slow in the early years, the number of publications rose steadily from 1980 to 2000. After 2000, despite fluctuations in publication volume (2001–2020), the overall total continued to rise significantly (Fig. 2B).

### 3.2. Analysis of countries

**Figure 3.**
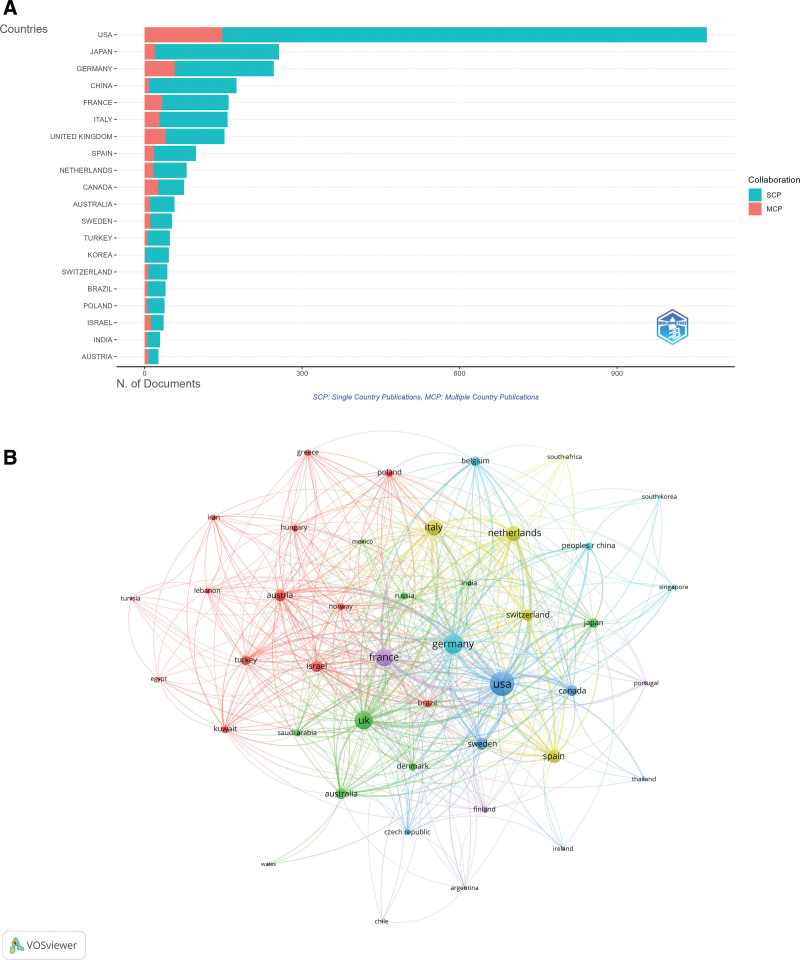
Country-level research contributions and collaboration. (A) Geographic distribution of corresponding authors by country. (B) Network map of international collaborations among countries; node size indicates publication volume, and line thickness reflects collaboration strength. (Source: Web of Science Core Collection; VOSviewer).

The contribution of the USA to this field was undisputed, with a total publication (TP) count of 4228, 148 multi-country collaborative publications, a total citation count (TC) of 58,055, and an average citation frequency of 54.2, all of which are the highest in the field. In addition, Germany (TP = 1138, TC = 11,935) and Japan (TP = 1025, TC = 6576) also made significant contributions to the field (Table 1 and Fig. 3A). In the collaboration network of 45 countries with at least 5 publications involved in international cooperation, the USA served as the central hub of global research, with a link strength of 549, ranking first. Germany followed closely with a link strength of 362, while the UK ranked third with a link strength of 319 (Fig. 3B).

**Table 1:** Publication and citation profiles of leading countries.

Country	Articles	Freq	MCP-Ratio	TP	TP-rank	TC	TC-rank	Average Citations
USA	1071	0.330	0.138	4228	1	58,055	1	54.2
Japan	256	0.079	0.078	1025	3	6576	4	25.7
Germany	246	0.076	0.236	1138	2	11,935	2	48.5
China	175	0.054	0.046	573	8	2766	8	15.8
France	160	0.049	0.206	942	4	6270	5	39.2
Italy	158	0.049	0.177	683	5	4999	6	31.6
United kingdom	152	0.047	0.263	640	6	7686	3	50.6
Spain	98	0.030	0.184	591	7	2450	10	25
Netherlands	80	0.025	0.213	320	10	3875	7	48.4
Canada	75	0.023	0.347	334	9	2083	12	27.8
Australia	57	0.018	0.175	310	11	1938	13	34
Sweden	52	0.016	0.212	253	12	2666	9	51.3
Turkey	48	0.015	0.104	204	14	414	22	8.6
Korea	46	0.014	0.022	165	16	665	16	14.5
Switzerland	43	0.013	0.163	235	13	2148	11	50
Brazil	40	0.012	0.150	176	15	484	18	12.1
Poland	38	0.012	0.132	162	17	393	23	10.3
Israel	36	0.011	0.333	100	21	1109	14	30.8
India	29	0.009	0.172	88	23	367	24	12.7
Austria	26	0.008	0.269	146	18	805	15	31

Articles: publications of corresponding authors only.

Average Citations = The average number of citations per publication, Freq = frequence of total publications, MCP-Ratio = proportion of multiple country publications, TC = total citations, TC-rank = rank of total citations, TP = total publications, TP-rank = rank of total publications.

### 3.3. Analysis of institutions

**Figure 4.**
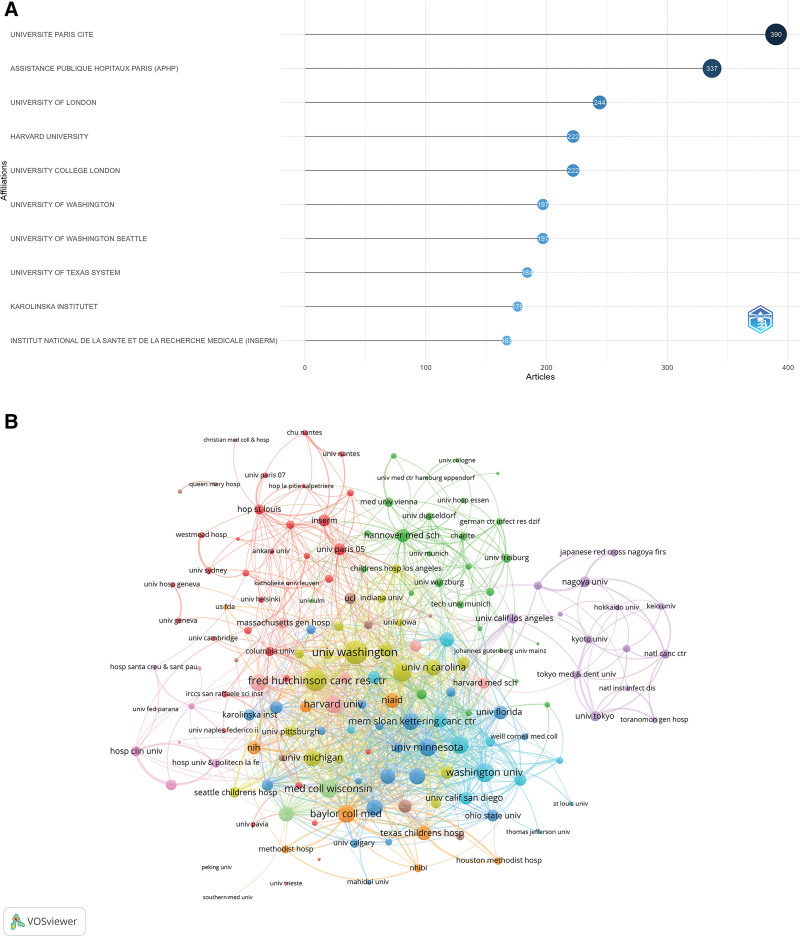
Institutional productivity and collaboration. (A) Top ten institutions ranked by number of publications. (B) Network map of inter-institutional collaboration; node size represents publication volume, and line thickness indicates collaboration strength. (Source: Web of Science Core Collection; VOSviewer).

Among the 11,858 institutions publishing relevant articles, the top 3 institutions by publication volume were: Université Paris Cité (390 papers), Assistance Publique – Hôpitaux de Paris (APHP) (337 papers), and University College London (244 papers) (Fig. 4A).

A total of 163 institutions published at least 5 international collaborative papers. The collaboration network map showed that the University of Washington was at the center of the cluster, with a link strength of 203, ranking first. Following closely are Fred Hutchinson Cancer Center with a link strength of 169, in second place, and Duke University with a link strength of 124, ranked third (Fig. 4B).

### 3.4. Analysis of Journals

The top 20 high-impact journals ranked by H-index were presented in Table 2. Among these journals, *Blood* exhibited the highest H-index of 74, accompanied by an IF of 21 and a TC of 11,579, both of which rank first in their respective categories. It also ranked third in TP volume, with 132 papers. The second-ranked journal was *Bone-Marrow Transplantation*, which has an H-index of 53, a TP volume of 221 papers (ranking first), and a TC count of 6852.

**Table 2:** Bibliometric indicators of high-impact journals.

Journal	H-index	IF	JCR-quartile	PY-start	TP	TP-rank	TC	TC-rank
Blood	74	21	Q1	1989	132	3	11,579	1
Bone-Marrow Transplant	53	4.5	Q1	1986	221	1	6852	2
Biol Blood Marrow Transplant	45	N/A	N/A	2000	142	2	3765	5
J Virol	43	4	Q2	1993	71	7	3851	4
Transplantation	37	5.3	Q1	1981	73	6	3045	7
Clin Infect Dis	33	8.2	Q1	1996	41	14	3006	8
Br J Haematol	30	5.1	Q1	1994	43	13	1755	14
J Infect Dis	27	5	Q1	1983	47	11	3288	6
J Immunol	26	3.6	Q2	1981	45	12	2481	9
J Med Virol	26	6.8	Q1	1986	73	5	1211	17
Transpl Infect Dis	26	2.6	Q2	2005	105	4	1096	18
J Clin Microbiol	24	6.1	Q1	1987	38	15	1915	12
J Clin Virol	23	4	Q2	1998	52	8	906	23
Am J Transplant	20	8.9	Q1	2003	29	19	1094	19
Hum Gene Ther	19	3.9	Q2	1992	28	22	544	36
Mol Ther	19	12.1	Q1	2001	26	23	698	28
Plos One	17	2.9	Q1	2007	36	17	634	29
Cytotherapy	15	3.7	Q2	2001	28	21	N/A	>50
Antimicrob Agents chemother	14	4.1	Q1	1997	18	30	573	33
Pediatr Transplant	14	1.2	Q3	2002	50	9	405	45

H-index: The h-index of the journal, which measures both the productivity and citation impact of the publications.

Average Citations = The average number of citations per publication, IF = impact factor, indicating the average number of citations to recent articles published in the journal, JCR-Quartile = the quartile ranking of the journal in the Journal citation reports, indicating the journal’s ranking relative to others in the same field (Q1: top 25%, Q2: 25%-50%, Q3: 50%-75%, Q4: bottom 25%), PY-start = publication year start, indicating the year the journal started publication, TC = total citations, TC-rank = rank of total citations, TP = total publications, TP-rank = rank of total publications.

**Figure 5.**
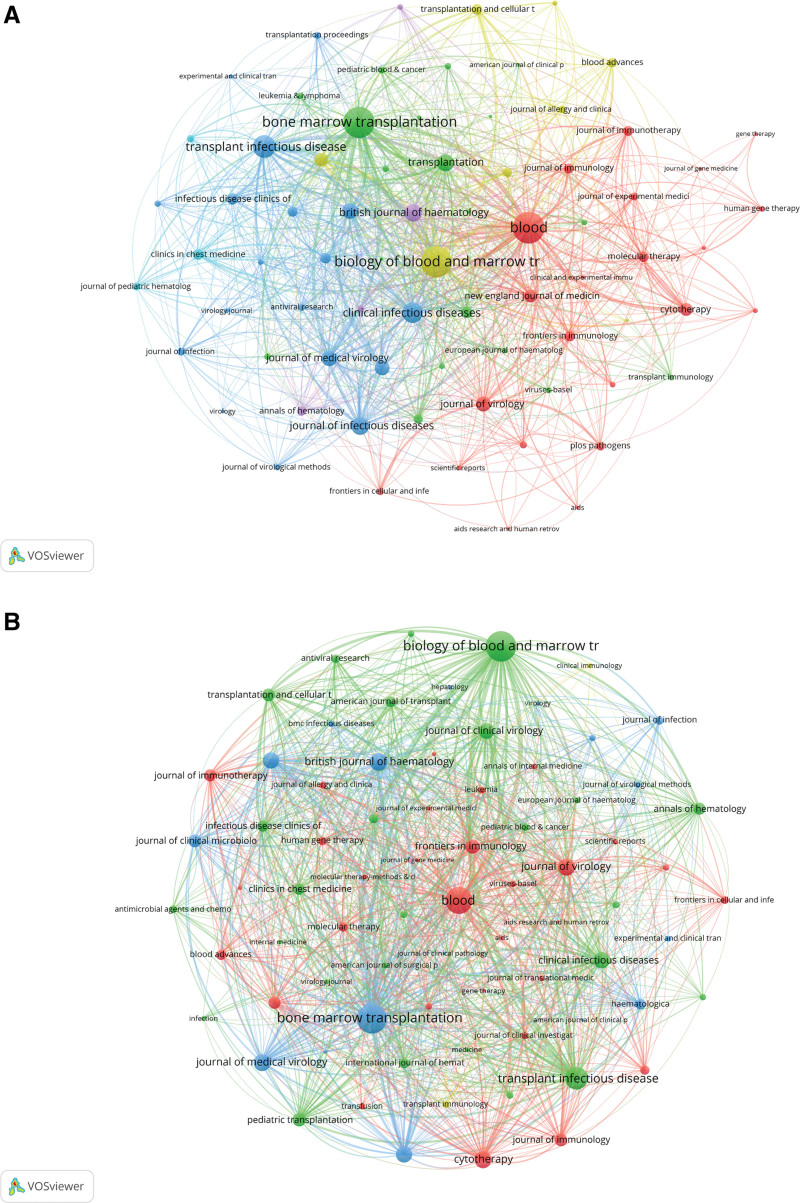
Journal network analysis. (A) Co-occurrence network highlighting journals publishing related research and their interconnections. (B) Coupling network displaying shared references among journals, indicating intellectual linkages. (Source: Web of Science Core Collection; VOSviewer).

In co-occurrence networks, which included 75 journals with at least 8 occurrences, the journals with the highest total link strength were *Blood* (1068), *Biology of Blood and Marrow Transplantation* (1001), and *Bone-Marrow Transplantation* (994) (Fig. 5A). In coupling networks, reflecting the extent of shared references among journals, the top 3 were *Biology of Blood and Marrow Transplantation* (total link strength 38,553), *Bone-Marrow Transplantation* (total link strength 37,852), and *Blood* (total link strength 30,868) (Fig. 5B).

### 3.5. Analysis of authors

As shown in Table 3, Boeckh Michael (H-index = 20, TP = 25, TC = 2729), Bollard Catherine M. (H-index = 19, TP = 34, TC = 2769), and Heslop Helen E. (H-index = 18, TP = 22, TC = 3353) were highly influential authors in this field.

**Table 3:** Publication and citation profiles of high-impact authors.

Authors	H-index	g-index	m-index	PY-start	TP	TP-Frac	TP-rank	TC	TC-rank
Boeckh michael	20	25	1.05	2006	25	2.66	2	2729	5
Bollard Catherine M.	19	34	1.12	2008	34	3.21	1	2769	4
Heslop Helen E.	18	22	0.95	2006	22	1.95	3	3353	1
Leen Ann M.	16	17	0.94	2008	17	1.57	8	2001	8
Chemaly Roy F.	15	17	0.79	2006	17	2.21	7	814	20
Rooney Cliona M.	15	16	0.88	2008	16	1.40	10	2984	3
Brenner Malcolm K.	14	18	0.74	2006	18	1.79	6	3206	2
Reddehase MJ	14	14	0.44	1993	14	2.61	16	1253	15
Veys Paul	14	16	0.74	2006	16	1.55	11	1148	16
Boeckh M	13	13	0.54	2001	13	1.86	18	2665	6
Einsele H	13	15	0.35	1988	15	2.02	12	537	38
Shpall Elizabeth J.	13	15	0.81	2009	15	1.00	13	1468	13
Hanley Patrick J.	12	19	0.75	2009	19	1.60	5	805	21
Socie Gerard	12	14	0.67	2007	14	1.14	17	543	37
Fischer A	11	12	0.34	1993	12	1.53	23	1053	17
Jahn G	11	13	0.34	1993	13	2.02	21	635	32
Navarro David	11	20	0.65	2008	20	1.76	4	404	43
Solano Carlos	11	17	0.65	2008	17	1.51	9	327	46
Amrolia Persis J.	10	11	0.53	2006	11	1.08	28	1567	12
Asano Y	10	10	0.33	1995	10	1.01	37	648	31

Average citations = The average number of citations per publication, g-index = The g-index of the journal, which gives more weight to highly-cited articles, H-index = The h-index of the journal, which measures both the productivity and citation impact of the publications, m-index = The m-index of the journal, which is the h-index divided by the number of years since the first published paper, PY-start = publication year start, indicating the year the journal started publication, TC = total citations, TC-rank = rank of total citations, TP = total publications, TP-rank = rank of total publications.

**Figure 6.**
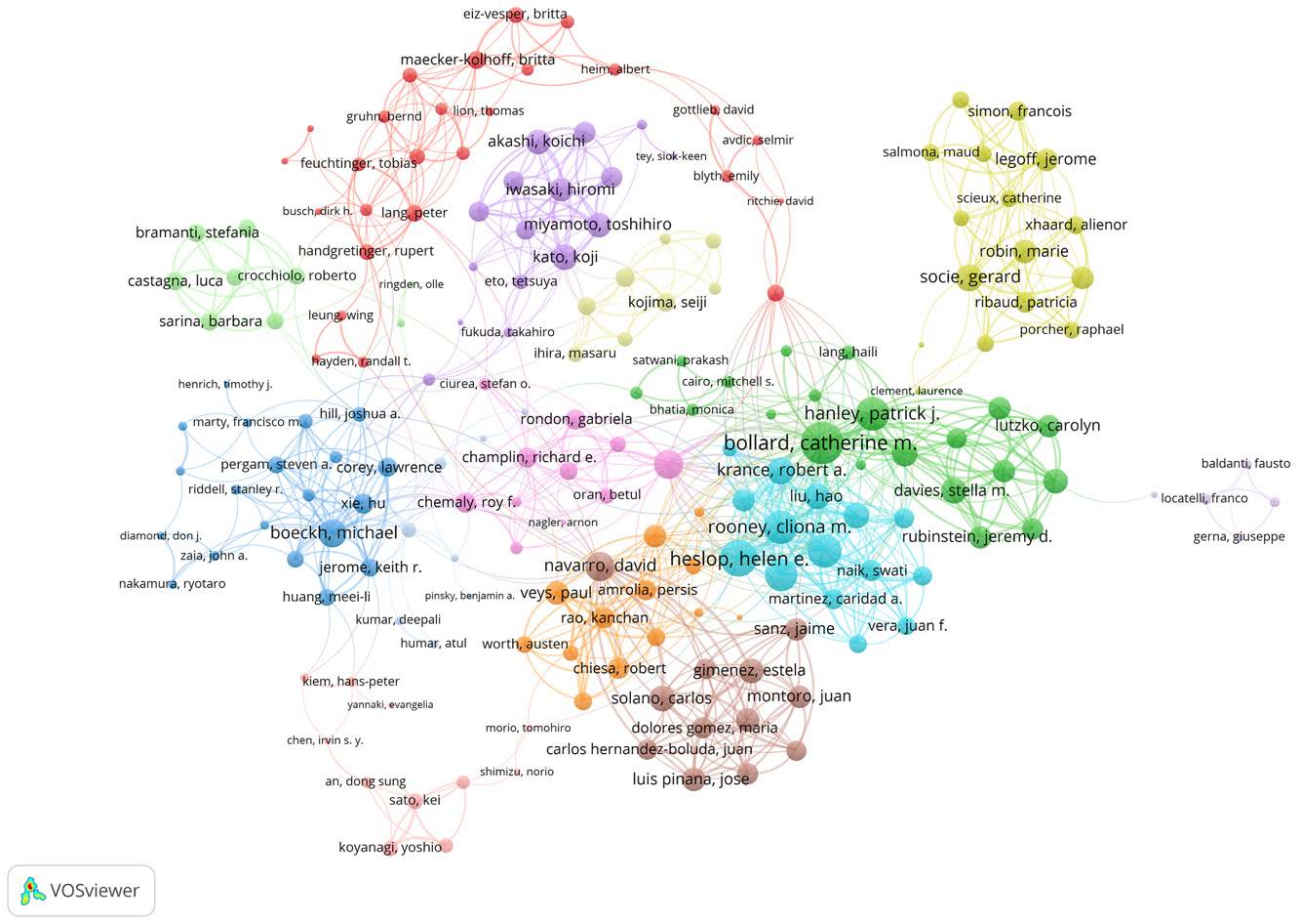
Author collaboration network. Visualization of author co-authorship; node size reflects publication output, and links represent collaborative relationships. (Source: Web of Science Core Collection; VOSviewer).

Among 350 authors with at least 5 international collaborations, Bollard Catherine M. led with total link strength of 158, followed by Heslop Helen E. (total link strength = 117) and Hanley Patrick J. (total link strength = 101), reflecting strong global partnerships (Fig. 6).

### 3.6. Keywords co-occurrence analysis

**Figure 7.**
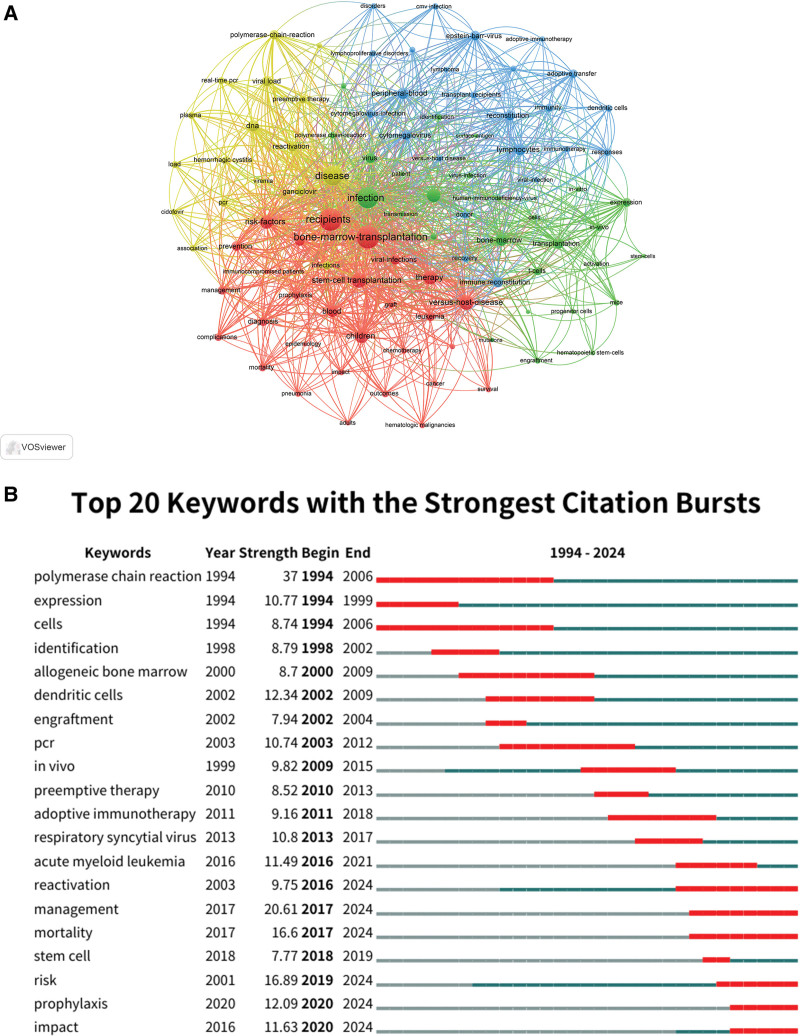
Keyword and trend analysis. (A) Co-occurrence network of keywords, grouped into thematic clusters. (B) Top 20 keywords with the strongest citation bursts, indicating emerging research hotspots. (Source: Web of Science Core Collection; VOSviewer and CiteSpace).

This study identified 143 keywords with a frequency of ≥ 30 occurrences and visualized their interrelationships using VOSviewer (Fig. 7A), forming 4 major thematic clusters representing core areas of research on viral-infections associated with HSCT: Cluster 1 (red, 29 items) focused on clinical outcomes and risk management, encompassing terms such as “bone marrow transplantation,” “children,” “chemotherapy,” “prevention,” “diagnosis,” “management,” “mortality,” “risk,” and “complications,” highlighting patient outcomes, risk-factors, and strategies for infection prevention and management in the HSCT context; Cluster 2 (green, 27 items) centered on basic science and mechanisms, including keywords like “activation,” “expression,” “infection,” “engraftment,” “progenitor cells,” and “replication,” emphasizing research on cellular mechanisms, molecular pathways, and transplantation biology; Cluster 3 (blue, 24 items) addresses immune response and immunotherapy, featuring terms such as “adoptive immunotherapy,” “d junioic cells,” “immune reconstitution,” “immunity,” “lymphocytes,” and “response,” reflecting exploration of posttransplant immune system reconstitution and immunotherapeutic strategies; Cluster 4 (yellow, 17 items) pertained to viral detection and monitoring, covering “polymerase chain reaction,” “real-time PCR,” “viral load,” “preemptive therapy,” “ganciclovir,” “cidofovir,” and “viremia,” indicating advancements in viral detection, diagnostic techniques, and therapeutic interventions. A detailed summary of the clusters and representative keywords is provided in Table 4.

**Table 4:** Keyword clustering analysis.

Cluster	Color	No. of keywords	Full keyword list
Cluster 1	Red	29	acute myeloid leukemia, adults, blood, bone-marrow-transplantation, cancer, chemotherapy, children, complications, diagnosis, epidemiology, graft, hematologic malignancies, immunocompromised, impact, leukemia, management, mortality, outcomes, pneumonia, prevention, prophylaxis, recipients, risk, risk-factors, stem-cell transplantation, survival, therapy, versus-host-disease, viral-infections
Cluster 2	Green	27	activation, bone-marrow, bone-marrow transplantation, cells, central-nervous-system, engraftment, expression, hematopoietic stem-cells, human-immunodeficiency-virus, identification, in vitro, infection, liver-transplantation, mice, mutations, patient, polymerase-chain reaction, progenitor cells, replication, stem-cells, surface-antigen, t-cells, transmission, virus, virus-infection
Cluster 3	Blue	24	adoptive immunotherapy, adoptive transfer, allogeneic bone-marrow, cmv infection, cytomegalovirus, cytomegalovirus-infection, dendritic cells, disorders, donor, epstein–barr-virus, high-risk, immune reconstitution, immunity, immunotherapy, lymphocytes, lymphoma, lymphoproliferative disorders, peripheral-blood, reconstitution, recovery, responses, transplant recipients, versus-host disease, viral-infection
Cluster 4	Yellow	17	association, cidofovir, disease, dna, ganciclovir, hemorrhagic cystitis, infections, load, pcr, plasma, polymerase-chain-reaction, preemptive therapy, reactivation, real-time pcr, renal-transplantation, viral load, viremia

### 3.7. Keyword burst analysis

To further elucidate research trends and emerging hotspots, we conducted a keyword burst analysis using CiteSpace (Fig. 7B). The top 20 keywords with the strongest citation bursts from 1994 to 2024 were identified and ranked by burst strength. The most pronounced burst was observed for “polymerase chain reaction” (strength = 37, 1994–2006), underscoring the impact of PCR in viral diagnostics during that period. Other notable bursts included “management” (strength = 20.61, 2017–2024), “risk” (strength = 16.89, 2001–2024), and “mortality” (strength = 16.6, 2017–2024), reflecting recent and ongoing attention to clinical management and patient outcomes. Early bursts were seen in keywords like “expression” (1994–1999), “identification” (1998–2002), and “dendritic cells” (2002–2009), corresponding to foundational research in immune response and viral detection. In recent years, “reactivation,” “prophylaxis,” “impact,” and “management” have emerged as sustained research frontiers, highlighting a shift towards proactive intervention and long-term patient care.

## 4. Discussion

This bibliometric analysis reveals several evolving research hotspots in the field of HSCT and viral-infections from 1980 to 2024. The co-occurrence and burst analyses indicate that early research focused on fundamental mechanisms such as gene expression, immune cell responses, and the development of viral detection technologies. Over time, the thematic emphasis has shifted toward clinical management, including risk assessment, prophylaxis, outcomes, and mortality among HSCT recipients. Notably, recent years have seen an increasing focus on viral reactivation, individualized management strategies, and long-term patient prognosis, reflecting both the growing complexity and precision of posttransplant care. These trends suggest that future research will likely prioritize the integration of advanced monitoring, immunotherapy, and personalized prevention approaches to further improve HSCT outcomes and reduce the burden of viral complications.

The USA exhibited a leading position in publication volume, with major institutions such as the University of Washington and Harvard University making substantial contributions. Significant funding from agencies such as the National Institutes of Health, alongside robust governmental support in the USA, facilitated comprehensive research initiatives, large-scale clinical trials, and a collaborative research network that spans North America.^[27,28]^ Similarly, notable scholars such as Catherine M. Bollard, also based in the USA, have advanced the field of pediatric immunology research significantly.^[29]^ Prominent journals in this discipline include *Bone-Marrow Transplantation*, *Biology of Blood and Marrow Transplantation*, and *Blood*, with *Blood* achieving the highest rankings in citations and impact factor (IF). These journals have published a significant number of influential articles that contribute to both the fundamental research and clinical applications of HSCT, as well as the pioneering exploration of innovative research on immune reconstitution and preventive strategies for viral-infections. This body of work has facilitated rapid advancements in the field and enhancements in clinical practice.^[30]^

Recent literature has also highlighted the importance of autophagy in HSCT, which plays a crucial role in maintaining hematopoietic stem-cell function and modulating immune responses during transplantation.^[31]^ In addition, the differentiation potential of HSCs toward NK cells is influenced by various cytokines and signaling pathways, further impacting immune reconstitution and infection risk in HSCT recipients.^[32,33]^ Such insights underscore the expanding scope of research that now encompasses not just virological and immunological mechanisms but also the broader cellular and molecular landscape of HSCT.

### 4.1. Research hotspots and frontiers

The keyword clustering analysis not only maps the intellectual structure of HSCT and viral-infection research, but also highlights the evolution from basic science to clinical management and technological innovation. Here, we systematically discuss each major cluster, integrating thematic focus, clinical implications, and key literature.

### 4.2. Cluster 1: clinical outcomes and risk management

This cluster centers on terms such as “acute myeloid leukemia,” “bone marrow transplantation,” “management,” “mortality,” “risk,” and “children,” reflecting the field’s persistent emphasis on patient-centered clinical outcomes, risk stratification, and posttransplant complications.

The risk of viral-infections in HSCT recipients is well-documented as a major contributor to non-relapse mortality, particularly among pediatric and immunocompromised populations.^[6,7]^ This is especially true for CMV, which remains one of the most clinically significant pathogens, with reactivation rates of up to 60% in allogeneic HSCT patients and associated mortality rates as high as 20%–35% in the absence of timely intervention.^[7,10,11]^ Recent studies highlight the utility of integrated risk models that incorporate clinical, virological, and immunological parameters to inform individualized management strategies.^[34]^ Such approaches are critical in pediatric transplantation, where children are more susceptible to viral complications and associated morbidity.^[12–14]^ Prophylactic and preemptive strategies, including antiviral agents and immunotherapy, have become standard components of clinical management, supported by evidence demonstrating improved patient outcomes and reduced viral disease burden.^[7,35,36]^

### 4.3. Cluster 2: basic science and mechanisms

Cluster 2 encompasses “activation,” “expression,” “engraftment,” “infection,” “progenitor cells,” “replication,” and “stem-cells,” underscoring research focused on the molecular and cellular mechanisms underlying immune reconstitution and viral pathogenesis.

The reconstitution of both innate and adaptive immunity following HSCT is a critical determinant of infection risk. Early work in this area elucidated the role of gene expression in modulating antiviral responses, providing the foundation for subsequent advances in personalized immunotherapy.^[1,37,38]^ The process of engraftment and hematopoietic recovery is influenced by factors such as stem cell source, conditioning regimen, and host genetics, each of which can also modulate susceptibility to viral-infections.^[2,3]^ Experimental models have shown that the expression and activation of specific immune cell subsets, including T-cells and dendritic cells, are pivotal in the control of viral replication and clearance.^[39,40]^ Recent research into autophagy has provided further insight into how this process supports the maintenance and function of HSCs during transplantation.^[31]^ Moreover, advances in our understanding of viral genome replication and cell tropism have yielded novel diagnostic and therapeutic targets, including the use of gene-edited progenitor cells and engineered immune effectors.^[1,29]^ This cluster highlights the continuous interplay between basic science and translational research, informing both the development of innovative therapies and the refinement of risk prediction models for clinical practice.^[1,40]^

### 4.4. Cluster 3: immune response and immunotherapy

The third cluster, with keywords such as “adoptive immunotherapy,” “dendritic cells,” “immune reconstitution,” “lymphocytes,” and “responses,” reflects a major thematic shift towards immunomodulatory approaches and precision medicine in managing viral complications post-HSCT.

Adoptive immunotherapy, particularly the infusion of virus-specific T-cells, has emerged as a promising strategy to restore antiviral immunity in HSCT recipients, especially those with refractory or recurrent viral-infections.^[29,39,40]^ Studies have demonstrated the effectiveness of ex vivo expanded or genetically modified T-cells in controlling CMV, EBV, and adenovirus infections, with minimal risk of graft-versus-host disease.^[29]^ Dendritic cells also play a critical role as antigen-presenting cells, bridging innate and adaptive immunity and influencing the efficacy of immunotherapeutic interventions.^[39,40]^ The differentiation of HSCs into NK cells, influenced by specific cytokines and microenvironmental factors, represents a parallel area of growing interest, as NK cells are pivotal in antiviral defense posttransplant.^[32,33]^ The dynamic monitoring of lymphocyte subsets and immune reconstitution kinetics has become an essential tool in predicting infection risk, guiding the timing of immunosuppressive tapering, and optimizing posttransplant care protocols.^[41,42]^ The integration of immunological biomarkers into clinical workflows represents a key advancement in personalized transplant medicine, enabling risk-adapted interventions and improved patient outcomes.^[5,29,30,43]^

### 4.5. Cluster 4: viral detection and monitoring

Cluster 4 includes “polymerase chain reaction,” “preemptive therapy,” “viral load,” “real-time PCR,” “viremia,” and “ganciclovir.” This cluster highlights the transformative impact of diagnostic technology and viral monitoring on early intervention and patient prognosis.

The adoption of molecular diagnostics, particularly quantitative PCR and real-time PCR, has revolutionized the management of viral-infections in HSCT recipients.^[10]^ These technologies allow for the rapid and sensitive detection of viral DNAemia, enabling preemptive therapy before the onset of symptomatic disease.^[10,35]^ For example, the monitoring of CMV viral load is now standard practice in most transplant centers, facilitating timely initiation of antiviral agents such as ganciclovir and reducing the incidence of end-organ disease.^[7,10,11]^ The refinement of monitoring protocols and the development of more specific assays for other pathogens (e.g., EBV, adenovirus, respiratory viruses) have further improved the capacity for early detection and intervention.^[12–14,44]^ As a result, preemptive therapy has become a cornerstone of posttransplant virological surveillance, contributing to significant reductions in infection-related morbidity and mortality.^[7,10,36]^

The analysis of citation bursts provides a dynamic perspective on the evolution of research priorities and the identification of emerging frontiers. Notably, recent years have witnessed sustained bursts in keywords such as “management,” “risk,” “mortality,” “prophylaxis,” “impact,” and “reactivation,” reflecting the field’s growing focus on comprehensive patient care and long-term outcomes. The prominence of “management” and “risk” as burst keywords underscores the shift from isolated infection control towards holistic, multidisciplinary strategies that address the multifactorial nature of posttransplant complications.^[5,34,45]^ The management of late viral reactivation and its consequences, such as increased relapse risk and non-relapse mortality in diseases like AML, has become a central research priority.^[34]^ Similarly, the keyword “prophylaxis” highlights the ongoing development and evaluation of preventive interventions, including novel antiviral agents, vaccine strategies, and immunoprophylaxis regimens.^[36,43]^ The increasing attention to “impact” and “mortality” reflects a broader recognition of the need to understand not only the immediate effects of viral-infections but also their long-term implications for immune health, quality of life, and overall survival.^[4,44,45]^

Importantly, these trends align with recent international consensus guidelines that advocate for risk-adapted, evidence-based protocols in HSCT recipient management, integrating virological monitoring, immunological assessment, and tailored therapeutic regimens.^[7,11,35]^ The convergence of clinical experience, technological innovation, and translational research is expected to drive the next generation of interventions, with a particular emphasis on early detection, individualized patient care, and the mitigation of late complications.^[1,4,30]^

### 4.6. Strengths and limitations

Several limitations should be acknowledged in this bibliometric analysis. First, the reliance on citation counts and bibliometric indicators may not fully capture the clinical relevance, scientific quality, or real-world impact of individual studies. Citation practices can be influenced by factors such as journal visibility, self-citation, and network effects, potentially introducing bias into the evaluation of research influence. Second, the analysis was limited to English-language publications indexed in select databases, which may have resulted in the omission of important studies published in other languages or in local journals, thereby restricting the global comprehensiveness and representativeness of the results. Third, bibliometric tools are constrained by the accuracy of keyword assignment and author affiliation data, which can affect the interpretation of research trends and collaboration networks. Additionally, this study primarily focused on published literature and did not include emerging research from preprints, ongoing clinical trials, or gray literature, which may also contribute valuable insights to the field. Finally, the rapidly evolving landscape of HSCT and viral-infection research means that some of the most recent developments may not yet be fully reflected in citation metrics or keyword analyses. These limitations should be considered when interpreting the findings and their implications for future research directions.

## 5. Conclusion

This bibliometric analysis comprehensively elucidates the research landscape at the intersection of HSCT and viral-infections. The findings highlight emerging frontiers in the field, focusing on risk stratification, personalized management, and innovative diagnostic and therapeutic approaches. These insights provide guidance for future research priorities, advocating the integration of advanced monitoring technologies, individualized immunotherapeutic strategies, and targeted preventive interventions to further improve outcomes and quality of life for HSCT patients facing viral complications.

## Author contributions

**Conceptualization:** Minjing Mao.

**Data curation:** Minjing Mao, Jiacheng Zhu.

**Formal analysis:** Jiacheng Zhu, Gang Cai.

**Funding acquisition:** Jun Meng.

**Investigation:** Jun Meng.

**Project administration:** Jun Meng.

**Resources:** Minjing Mao, Gang Cai.

**Software:** Minjing Mao.

**Supervision:** Jun Meng.

**Validation:** Jiacheng Zhu, Gang Cai.

**Visualization:** Jiacheng Zhu, Gang Cai.

**Writing – original draft:** Minjing Mao, Jiacheng Zhu, Gang Cai, Jun Meng.

**Writing – review & editing:** Minjing Mao, Jiacheng Zhu, Gang Cai, Jun Meng.
